# Monitoring the Progression of Cell-Free Expression of Microbial Rhodopsins by Surface Enhanced IR Spectroscopy: Resolving a Branch Point for Successful/Unsuccessful Folding

**DOI:** 10.3389/fmolb.2022.929285

**Published:** 2022-07-14

**Authors:** Kenichi Ataka, Axel Baumann, Jheng-Liang Chen, Aoife Redlich, Joachim Heberle, Ramona Schlesinger

**Affiliations:** ^1^ Department of Physics, Experimental Molecular Biophysics, Freie Universität Berlin, Berlin, Germany; ^2^ Department of Physics, Genetic Biophysics, Freie Universität Berlin, Berlin, Germany

**Keywords:** FTIR—spectroscopy, membrane protein folding, cell-free expression, SEIRA (surface enhanced infrared absorption), sensory rhodopsin, channelrhodopsin, co-translational folding

## Abstract

The translocon-unassisted folding process of transmembrane domains of the microbial rhodopsins sensory rhodopsin I (*Hs*SRI) and II (*Hs*SRII), channelrhodopsin II (*Cr*ChR2), and bacteriorhodopsin (*HsBR*) during cell-free expression has been investigated by Surface-Enhanced Infrared Absorption Spectroscopy (SEIRAS). Up to now, only a limited number of rhodopsins have been expressed and folded into the functional holoprotein in cell free expression systems, while other microbial rhodopsins fail to properly bind the chromophore all-*trans* retinal as indicated by the missing visible absorption. SEIRAS experiments suggest that all investigated rhodopsins lead to the production of polypeptides, which are co-translationally inserted into a solid-supported lipid bilayer during the first hour after the *in-vitro* expression is initiated. Secondary structure analysis of the IR spectra revealed that the polypeptides form a comparable amount of α-helical structure during the initial phase of insertion into the lipid bilayer. As the process progressed (>1 h), only *Hs*BR exhibited a further increase and association of α-helices to form a compact tertiary structure, while the helical contents of the other rhodopsins stagnated. This result suggests that the molecular reason for the unsuccessful cell-free expression of the two sensory rhodopsins and of *Cr*ChR2 is not due to the translation process, but rather to the folding process during the post-translational period. Taking our previous observation into account that *Hs*BR fails to form a tertiary structure in the absence of its retinal, we infer that the chromophore retinal is an integral component of the compaction of the polypeptide into its tertiary structure and the formation of a fully functional protein.

## Introduction

Membrane proteins are central targets in many modern drug developments (Wallin and Heijne, 1998) but their functional expression in heterologous hosts is still a challenging and formidable task. One of the major obstacles of membrane protein synthesis is the complex interplay between the nascent peptide chains and the cell membrane to yield a properly folded protein. To understand this process one has not only to clarify the folding process of the protein itself, but also consider the particular properties of the lipid environment (Cymer et al., 2015). The latter depends on the complex chemistry of the lipid molecules such as charge on the head groups, physicochemical specificity arising from head group size, packing-densities of the lipids, length of the hydrophobic chain, degree of saturated and unsaturated bonds, etc. (Findlay and Booth, 2006). Because of this complexity, the study of membrane protein folding is far behind recent research progress in the field of water-soluble proteins (Rose, 2021), in which the latter succeeded to predict even full protein structures from their amino acid sequence using computational methods (Tunyasuvunakool et al., 2021).

The recent development of cell-free expression is very useful for the study of membrane protein folding. Although the method itself has been known for decades (Eiserling et al., 1964), the overall synthesis efficiency was improved considerably by refinement of protocols and reaction designs (Henrich et al., 2015). This system has been successfully applied to studies of co-translational folding due to facile operation and large flexibility (Pellowe and Booth, 2020). For membrane proteins, cell-free expression is still a tedious trial and error process depending on the skills and practices of individual researchers. As of now, the expression of membrane proteins and their subsequent folding into the lipid bilayer is a complex biological process which cannot be easily monitored in each step. Evidently, it would be helpful to shine light into this black box by applying a method that is able to visualize individual processes during cell-free expression, insertion and folding to spot the progression of the process and where it fails (Harris et al., 2020). Means for variation of the expression strategy can be developed to successfully express the membrane protein of interest.

Surface-enhanced infrared absorption spectroscopy is an excellent tool to track secondary structure evolution during cell-free expression coupled with co-translational folding of a membrane protein (Baumann et al., 2016). We have applied this method to study the folding process of bacteriorhodopsin (*Hs*BR) during its production in a cell-free transcription/translation system. In our former study (Baumann et al., 2016), we have succeeded to trace the dynamics from the early insertion and folding of the nascent polypeptide chain into the membrane to the late formation of tertiary structure of the protein. Similar studies have been performed on two other membrane proteins, namely DsbB and the rhomboid protease GlpG, both α-helical proteins but with different number as compared to rhodopsins (Harris et al., 2017). A major result of these studies is that individual proteins folded into the lipid membrane correctly in the absence of a translocon, however, with different folding pathways.

Up to now, only a limited number of microbial rhodopsins like *Hs*BR (Katzen et al., 2008) and proteorhodopsin (Roos et al., 2012) were expressed in cell-free expression systems to yield functional transmembrane proteins immersed in lipid nanodiscs. Yet, many other types of rhodopsins are still not successfully expressed in cell-free system although such facile expression are demanded because of, for example, their application in optogenetics. Our approach is to study the folding of a protein when released from the ribosome during translation, which has a potential to elucidate factors that are decisive for a working expression system.

Here, we choose three microbial rhodopsins, which failed in an *in vitro* batch expression, as no specific visible absorption of the holoproteins was discernible. Sensory rhodopsin I and II from *Halobacterium salinarum* (*Hs*SRI and *Hs*SRII) are two prokaryotic proteins, which are expressed well in *E. coli* but obviously not in the *E. coli* based *in vitro* system. Channelrhodopsin-2 from *Chlamydomonas reinhardtii* (*Cr*ChR2) is the most used optogenetic tool with eukaryotic origin. Examination by SEIRA studies showed, however, that expression and translation of the proteins takes place but folding and reconstitution with the chromophore retinal into the lipid membrane is impaired. In this study, we report on the details of the insertion and folding of these microbial rhodopsins into solid-supported lipid bilayers and discuss the differences to the successful folding process of *Hs*BR.

## Materials and Methods

### Cell-Free Protein Expression

*E. coli*-based cell-free protein expression was set up using a MembraneMax™ HN Protein Expression Kit (Invitrogen^®^) according to the manufacturer’s description. The kit includes following major components: polyhistidine-tagged nanodiscs with DMPC (1,2-dimyristoyl-sn-glycero-3-phosphocholine) lipid bilayer, an optimized *E. coli* slyD^−^ extract, reaction buffer containing an ATP regeneration system, a feed buffer to replenish components, amino acids and T7 RNA polymerase. The all-*trans*-retinal was supplied from a 10 mM stock solution in ethanol and not taken from the kit.

For the studies of *Hs*BR, the plasmid pEXP5-CT/bR encoding bacterioopsin gene (*bop*), provided as a part of the kit, was used for cell-free protein expression. The plasmids pET 27b/Chop2, pEXP5-CT/SRI, pEXP5-CT/SRII, which encode the apoproteins for *Cr*ChR2_1–307_, *Hs*SRI and *Hs*SRII, respectively, were used for the cell-free expression. The coding sequence for the transmembrane part of *Cr*ChR2_1–307_ (Krause et al., 2013) with a C-terminal extension of ASHHHHHH including a 6xHis tag was cloned into pET 27b between the *Nde*I and *Hind*III sites. *Hs*SRI with a 10xHis tag (Mironova et al., 2007) and *Hs*SRII with a 6xHis tag (Mironova et al., 2005) were cloned into pEXP5-CT vector by substituting the *bop* gene.

### UV/Visible Spectroscopy

A microliter cuvette cell with a cell lid with 2 mm of light path length (Implen NanoPhotometer^®^) was mounted onto UV/visible spectrometer (Shimadzu UV-2600i). The samples were first centrifuged at 20,800 × g for 10 min to remove precipitation. 5 µl of each sample were applied. Each spectrum was measured with 2 s of accumulation time, 0.5 nm of resolution with slid width of 1 nm.

### Surface Enhanced Infrared Spectroscopy

The experimental set up and procedures for SEIRAS have been described elsewhere (Ataka and Heberle, 2007). Briefly, a thin gold film was formed on the reflection surface of a triangular silicon prism by chemical deposition. The prism was mounted on the home-made Attenuated Total Reflection (ATR) optics with a plexiglas cell to hold the sample solution. With this configuration, the reaction solution can be easily added or exchanged to the sample during the IR measurement. All infrared spectra were measured with Bruker Vertex 70v spectrometer (Bruker Optik GmbH, Ettlingen Germany) equipped with MCT detector. The peak fitting and other data treatment of the recorded spectra are described in SI-2 and were handled by the program IGOR (WaveMatrics Inc.).

### *In-Situ* Measurement of the Cell-Free Expression

The procedure of the *in-situ* measurement of cell-free expression is described in detail elsewhere (Baumann et al., 2016). A self-assembled monolayer of Ni-nitrilotriacetic acid (NTA) was formed on a gold film surface. On top of the Ni-NTA coated gold surface, a monolayer of nanodiscs with DMPC lipid bilayer was coupled *via* the 6xHis-tag at the N-terminus of the membrane scaffold proteins. After the formation of the nanodiscs monolayer, the surface was incubated with the reaction mixture containing all components for the cell-free expression except the plasmid DNA of the individual target membrane protein. After about 1 h of incubation time, expression of the sample protein is initialized by adding the target plasmid to the feeding mix. The diameter of a nanodisc is approximately 10 nm. Under the conditions set here, it is presumed that not more than a single ribosome of ca. 20 nm in diameter can bind to each nanodisc. The time-resolved IR measurement starts simultaneously with the addition of the plasmid DNA. All FTIR spectra were recorded at room temperature (25°C).

## Results

### UV/Vis Spectroscopy of *In Vitro* Translated Microbial Rhodopsins

**FIGURE 1:**
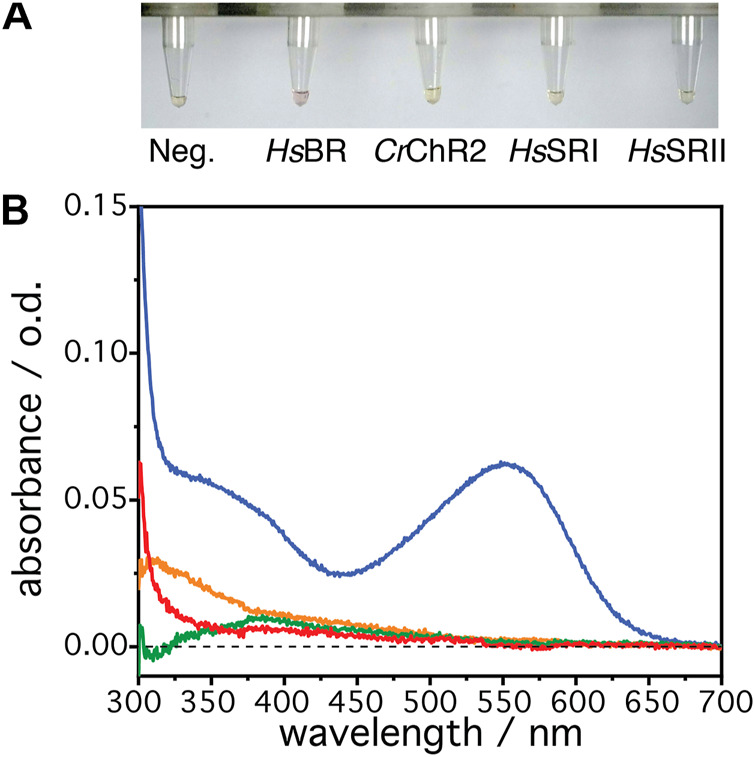
**(A)** The resultant solutions from the cell-free expression at 25°C, 8 h after the addition of the individual DNA plasmid. From left to right: Negative control (no DNA), bacteriorhodopsin (*Hs*BR), channelrhodopsin II (*Cr*ChR2), sensory rhodopsin I (*Hs*SRI), and sensory rhodopsin II (*Hs*SRII). **(B)** UV/visible absorption spectra of the resultant solutions of *Hs*BR (blue), *Hs*SRI (green), *Hs*SRII (red), and *Cr*ChR2 (orange). The solution with the negative control was used for the reference spectrum to compensate absorption from the free retinal in the solution and other components.

The microbial rhodopsins *Hs*BR, *Hs*SRI, *Hs*SRII and *Cr*ChR2 have been expressed in a parallel batch approach with a cell-free *E. coli* system. The expressions were handled at room temperature (25°C) in order to make direct comparison with IR data and at optimal 37°C. Lipid nanodiscs, dissolved in the batch solution, serve as a mimic of the native membrane and act as the target for the insertion and folding process. Figure 1A shows batch samples resulting from the cell-free expression trials for each of the four microbial rhodopsins at 25°C. Only the expression mixture for *Hs*BR shows slight red-pink color indicating successful reconstitution of the all-*trans* retinal chromophore into the core of the protein leading to a functional rhodopsin. The other mixtures remain slightly yellow, which is comparable to the negative control in which translation was not started due to the absence of a transcribable gene. The yellow color attests to free all-*trans* retinal added to the sample solution. In the case of successful reconstitution, a shift in the absorption should be visible with expected absorption maxima of ∼560 nm for *Hs*BR, ∼590 nm for *Hs*SRI, ∼490 nm for *Hs*SRII, and ∼470 nm for *Cr*ChR2, respectively (Ernst et al., 2014) which indicate bound chromophores to the retinal pocket. In Figure 1B, the UV/Vis spectra of the clear translation mixtures in the range of 300–700 nm are shown. The reference solution in the spectroscopic measurement was the negative expression control, where no expression was initialized by DNA, but which contained the same amount of free retinal and all other components. This led to a subtraction of the chromophore band, which has an absorption at ∼380 nm. The expression mixture with *Hs*BR (Figure 1B, blue spectrum) shows an absorption band at 560 nm, suggesting that *Hs*BR has been successfully expressed and subsequently correctly integrated its chromophore. In all other cases, no specific retinal signals can be detected suggesting that the chromophore was not incorporated into the apo-proteins of *Hs*SRI, *Hs*SRII, and *Cr*ChR2. These differences in the results of cell-free expression between *Hs*BR and other microbial rhodopsins become more obvious when they were expressed at 37°C, where the cell-free expression yield is optimized according to the manufacture’s description (Supplementary Information S1; Supplementary Figure S1). From these data, it cannot be concluded whether there is a problem in reconstitution of the protein or if the expressions failed already at the level of transcription and translation. As functional expression of *Hs*SRI, *Hs*SRII has been reported in *E. coli* (Schmies et al., 2000; Mironova et al., 2005; Mironova et al., 2007)*,* it was not clear why transcription/translation should give here no results in the cell-free expression based on *E. coli* extract. Interestingly, cell-free expression works for *Hs*BR quite well although the same gene gave no functional expression in living *E. coli* cells (data not shown). These results are puzzling as all these proteins have a similar arrangement of their seven transmembrane α-helices. Thus, the question arises: at which stage does functional expression of *Hs*SRI, *Hs*SRII, and *Cr*ChR2 fail? We approach this complex problem by recording *in-situ* IR spectra during expression and co-translational folding into a lipid membrane. As IR spectra provide structural information, we can derive information on whether the nascent apoproteins are properly produced, and whether they fold into proper secondary or higher structures.

### *In-Situ* Surface Enhanced Infrared Spectroscopy Monitoring Cell-Free Expression of Microbial Rhodopsins

**FIGURE 2:**
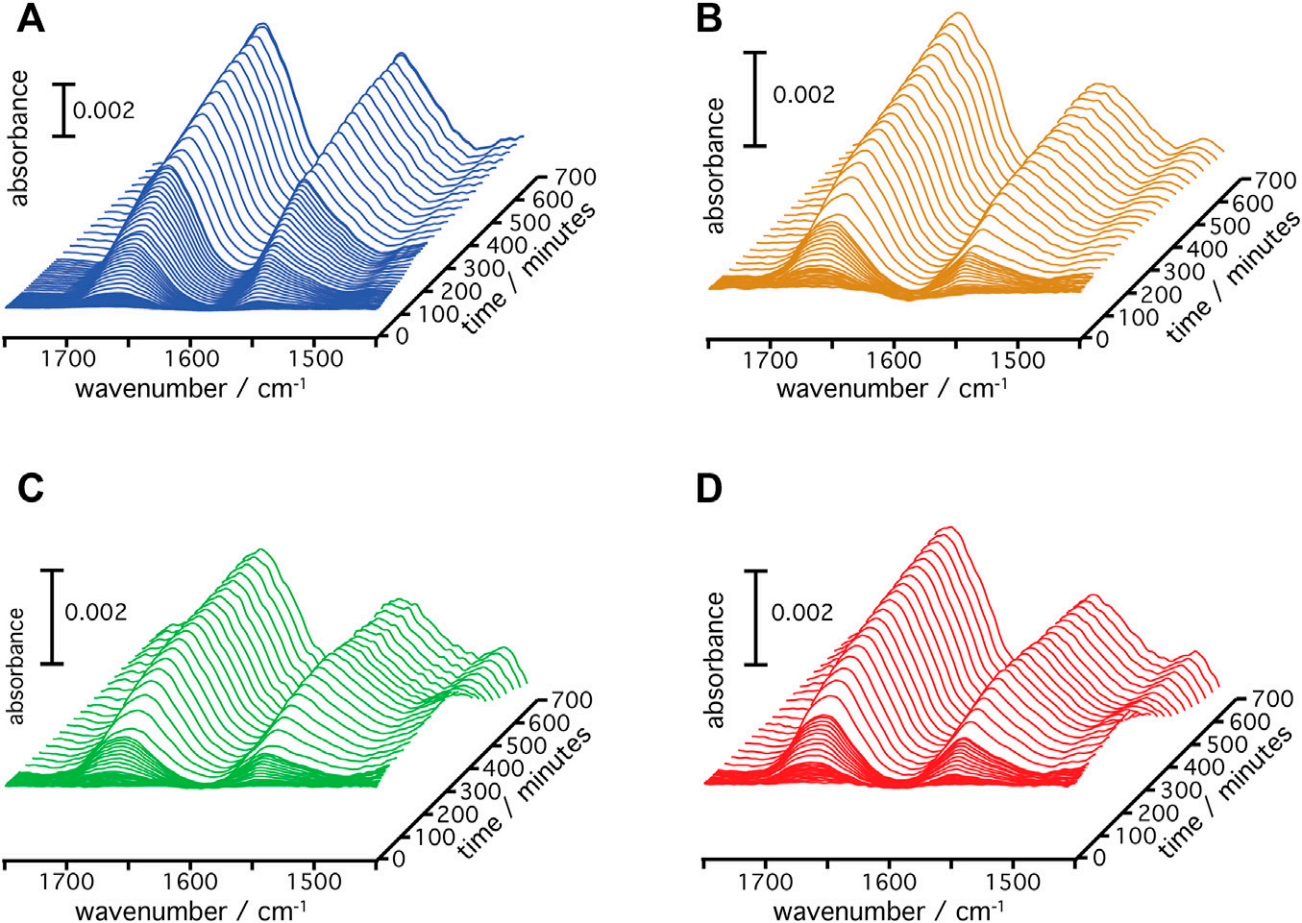
Time-resolved SEIRA spectra of **(A)** *Hs*BR, **(B)** *Cr*ChR2, **(C)** *Hs*SRI, and **(D)** *Hs*SRII during the cell-free expression and insertion into the DMPC nanodiscs are shown.

**FIGURE 3:**
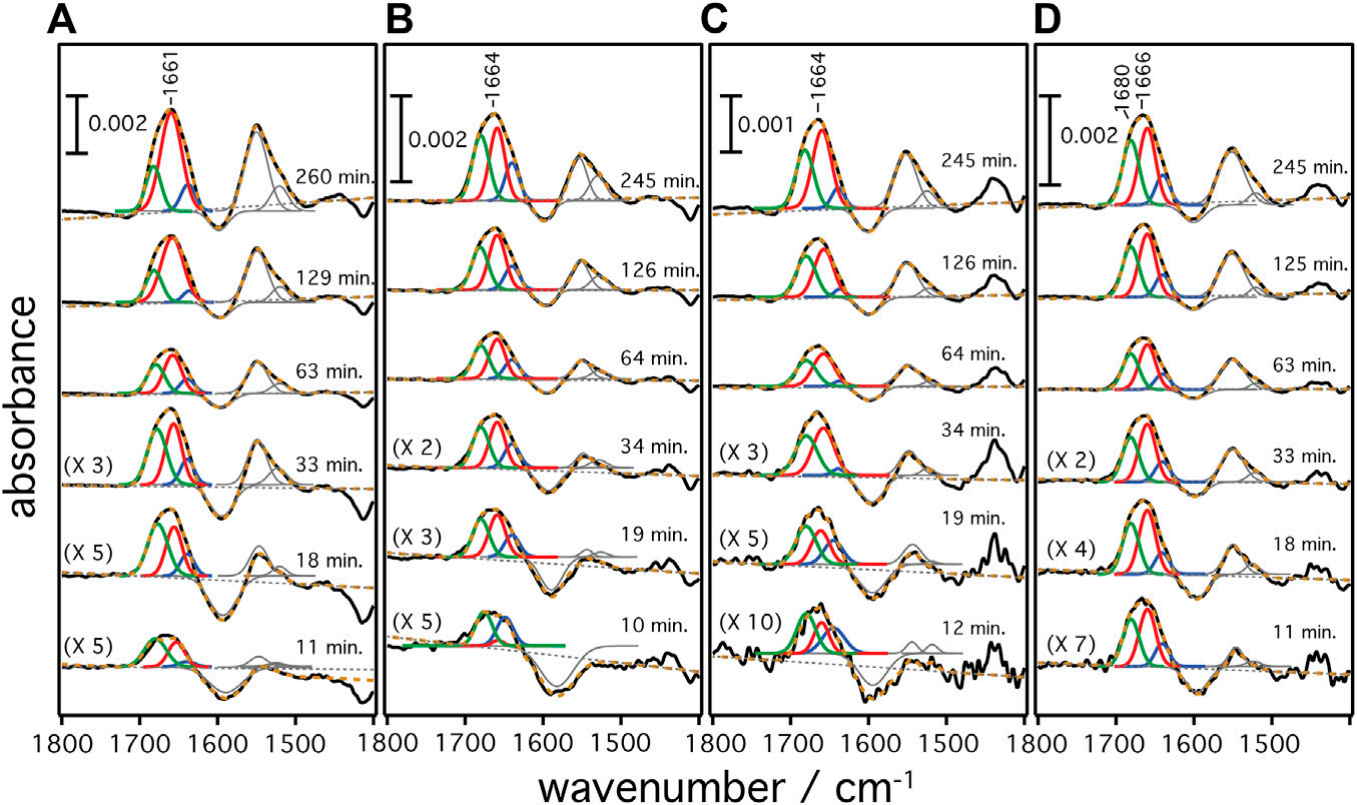
Secondary structure contributions over time during the cell-free expression of **(A)** *Hs*BR, **(B)** *Cr*ChR2, **(C)** *Hs*SRI, and **(D)** *Hs*SRII are given. Raw SEIRA spectra are shown as black solid curves. The dashed orange curves are the cumulative fit of the individual components. Colored fittings represent the contribution from bend/misfolded (green), α-helices (red), and random (blue) components, respectively.

A gold film was prepared atop a silicon prism onto which a layer of Ni-NTA was covalently linked bearing a monolayer of DMPC nanodiscs (Jiang et al., 2008). This surface was equilibrated with the cell-free expression mixture until spectra did not show any changes. Then, transcription and translation were triggered by addition of the target gene of the respective rhodopsin (*Hs*BR, *Hs*SRI, *Hs*SRII, or *Cr*ChR2) and spectra were recorded in a time-resolved manner (Figure 2). Irrespective of the microbial rhodopsin chosen, the recorded *in-situ* SEIRA spectra showed two characteristic bands appearing at around 1,661–1,666 cm^−1^ and 1,549–1,552 cm^−1^ after addition of the plasmid DNA. These bands are assigned to the amide I and II vibrational modes, respectively, of the nascent polypeptide backbone which bear mostly C=O stretching and coupled C=N stretching and N-H bending characters (Krimm and Bandekar, 1986; Barth, 2007). As these bands are clearly visible in all cases, it is evident that the nascent polypeptides appear close to the surface-attached nanodiscs because of the short range of the surface enhancement exploited by SEIRAS (Baumann et al., 2016). Close inspection of the individual spectra revealed that the peak positions of the amide I bands are slightly different. In the case of *Hs*BR, the amide I band peaks at 1,661 cm^−1^ (Figure 3A). This frequency is characteristic of properly folded *Hs*BR. It should be noted that the frequency of the amide I band of *Hs*BR is exceptionally higher than that of normal α-helical proteins (usually between 1,645 and 1,657 cm^−1^) due to strong vibrational coupling by forming a condensed bundle of transmembrane α-helices (Karjalainen and Barth, 2012). The final peak positions of the amide I bands of *in-vitro* expressed *Cr*ChR2, *Hs*SRI, and *Hs*SRII are at 1,664, 1,664, and 1,666 cm^−1^ (Figures 3B–D), respectively. These peak positions are too high for properly folded α-helical proteins even if an effect from vibrational coupling is considered. It should be noted that this frequency range can be congested and may include several overlapping bands assigned to various secondary structures with different peak positions (Krimm and Bandekar, 1986). In the spectra of *Cr*ChR2, *Hs*SRI, and *Hs*SRII, a shoulder band is clearly observed at around 1,680 cm^−1^, which can be assigned to turn structures from misfolded components in the amide backbone (Krimm and Bandekar, 1986). A large contribution from this higher shoulder band is overlapped with the α-helix components at 1,650–60 cm^−1^. As a consequence, it leads the apparent peak position of overall amide I band to be slightly higher than the usual α-helical frequency, when all component bands are combined. This point becomes clear when the amide I bands are decomposed into components of individual secondary structure elements by the peak fitting procedure outlined below.

### Peak Fitting and Elucidation of the Secondary Structure Components

**FIGURE 4:**
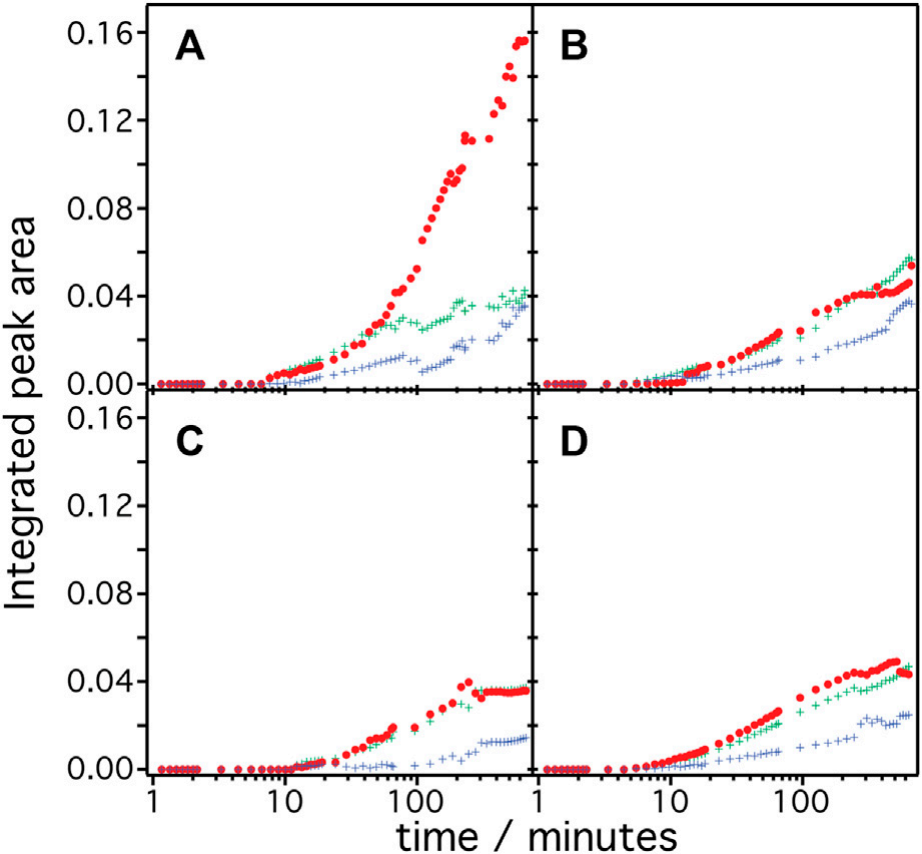
Integrated peak area of the secondary structure contribution over time during the cell-free expression of **(A)** *Hs*BR, **(B)** *Cr*ChR2, **(C)** *Hs*SRI, and **(D)** *Hs*SRII are shown. The color code represents the contribution from α-helices (red), bend/misfolded (green) and random (blue) components.

The frequency range between 1,700 and 1,600 cm^−1^ represents vibrational bands of the C=O stretching modes in the different secondary structures of the expressed polypeptides. As these bands are broad and overlapping, we applied peak fitting to identify the contributions of individual secondary structural elements (Figure 3). Fitting procedures were applied on the basis of three different secondary structures: 1) Turn/bend structures indicative of misfolded structure with weak hydrogen bonding among the backbone amides appearing in the range between 1,670 and 1,685 cm^−1^ (shown as green curves in panels A–D), 2) α-helical structures appearing between 1,645 and 1,661 cm^−1^ (shown as red curves), and 3) random and strongly hydrogen-bonded amide structures appearing between 1,635 and 1,645 cm^−1^ (shown as blue curves) (Barth, 2007). We exclude contributions from β-turns at >1,685 cm^−1^ or β-sheets/aggregates at <1,635 cm^−1^ because they are considered minor and are included as part of the components under (1) or (3). Details of the peak fitting procedures are described in the Supplementary Information S2 and Supplementary Figures S2–S5. The sum of the fitted peaks (orange broken curves in Figure 3) correspond well to the recorded spectra (black solid curve), indicating that fitting by three components works sufficiently well for spectra recorded at t > 10 min. In all samples, the amide bands rise at around 8–10 min after triggering transcription/translation by addition of DNA and increase in intensities over time. This result agrees well with the previously reported experiments on *Hs*BR (Baumann et al., 2016) indicating a conditioning period to remove non-specifically bound supramolecular species from the membrane surface before insertion of the nascent peptide chains takes place from the translating ribosome. Such a “pre-conditioning period” is equally observed in the case of *Cr*ChR2, *Hs*SRI, and *Hs*SRII, suggesting that peptide production and insertion of these microbial rhodopsins proceed similarly as with *Hs*BR as described previously (Baumann et al., 2016). The intensities of each structural component were plotted versus expression time (Figure 4) to reflect the kinetics of changes in secondary structure of the nascent polypeptides. From 10 min until approximately 60 min, intensities of each component increase monotonically over log-time for all proteins. After 60 min, only *Hs*BR shows an abrupt increase in its α-helical component (Figure 4A), while the other rhodopsins *Cr*ChR2, *Hs*SRI, and *Hs*SRII exhibit a slight but continuous increase in α-helical content along with the increase of the other components to reach saturation at about 5 h.

**FIGURE 5:**
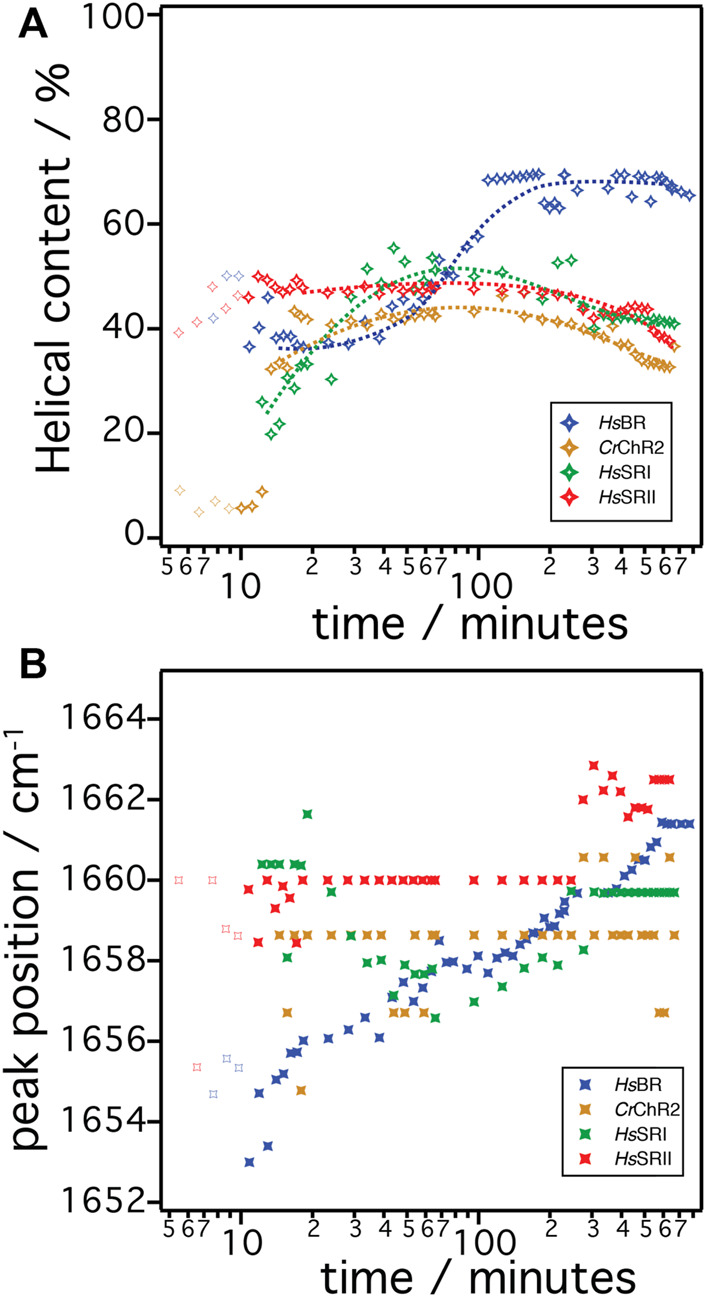
**(A)** The α-helical content in the amide I band over time during the cell free expression is shown. The helical content is determined by dividing the peak area of the α-helical component by the peak area of the cumulative of all components in amide I band. **(B)** The peak position of the α-helical components are plotted over time during the cell free expression. The color code represents *Hs*BR (blue), *Cr*ChR2 (orange), *Hs*SRI (green), and *Hs*SRII (red).

Since microbial rhodopsins consist predominantly of transmembrane α-helical structures, determination of the overall contents of α-helices in the structures is a good indicator to derive the degree of successful folding of the *in-vitro* produced peptide chain (Figure 5A). These are determined by dividing the integral of the α-helical component by the total of the amide I band (sum of all components). In the case of *Hs*BR (blue), the helical content is around 37% after 10–40 min. For *Cr*ChR2 (orange), *Hs*SRI (green), and *Hs*SRII (red), the corresponding values are between 40%–50% in the same time range. This suggests that the initial phase of helix formation equally works in the four proteins. However, the helical content of *Hs*BR abruptly increased at >50 min to ∼70% at 200 min. This sudden increase is only observed in *Hs*BR and not observed for other rhodopsins. We infer, based on our previous experiments (Baumann et al., 2016; Harris et al., 2017) that co-translational peptide production and membrane insertion of the nascent chain takes place during 10–60 min after induction of expression which is accompanied by the formation of α-helices. Within this period, the helical content stays at intermediate values around 40%–50% for all samples. While the helical proportion of the protein is constant, the amide band intensities continue to rise. This suggests that the produced nascent polypeptide immediately folds into secondary structure during the co-translational period to maintain a constant proportion of α-helical structure. Between 1–4 h, tertiary structure formation takes place in which the produced α-helices aggregate to form helical bundles (Baumann et al., 2016). This interpretation fits to the observed increase in helical content of *Hs*BR, as the formation of helical bundles leads to vibrational coupling resulting in a stronger transition dipole moment and a consequent higher IR absorption coefficient of the amide I mode.

The concept of secondary structure formation at 10–60 min and tertiary structure formation at >60 min is further supported by monitoring the shift of the peak position of the α-helical component (Figure 5B) (Karjalainen and Barth, 2012). The α-helical component of *Hs*BR (blue symbols in Figure 5B) exhibits a significant shift of the peak position from 1,653 cm^−1^ to 1,661 cm^−1^ during expression. Note that the shift occurs more drastically during the first hour as time is plotted on the log scale. It has been suggested that the α-helical amide I band shifts to higher wavenumbers when the peptide is exposed to a more hydrophobic environment (Chirgadze and Brazhnikov, 1974; Venyaminov and Kalnin, 1990). This happens during coupled secondary structure formation and the insertion of the nascent peptide into the membrane. After 60 min, the peak position stays at 1,658 cm^−1^ until 90 min, then starts to shift up again to higher wavenumbers. This restart of the upshift corresponds to the sudden rise of the intensities of the α-helical component (Figure 4A). This observation also fits to the interpretation of the vibrational coupling caused by the helical bundle formation, which affects both the intensity and peak position of the α-helical component (Karjalainen and Barth, 2012).

In contrast to the results on *Hs*BR, the other tested rhodopsins *Cr*ChR2, *Hs*SRI, and *Hs*SRII do not show drastic changes, neither in helical contents nor in peak positions after insertion and folding. The helical content of *Cr*ChR2 and *Hs*SRII remains almost constant at around 40% during expression, or even show slight decrease after 5 h. *Hs*SRI shows increase of the helical content up to ∼40% at times less than 1 h but decreases after 5 h like *Cr*ChR2 and *Hs*SRII. The peak positions of the α-helical components of *Cr*ChR2 and *Hs*SRII stay constant, while that of *Hs*SRI shows a slight down shift (3 cm^−1^) during 10–60 min after induction, then shift back to 1,660 cm^−1^ at 100 min and remain constant. Lack of these spectral changes in *Cr*ChR2, *Hs*SRI, and *Hs*SRII at >1 h is a major difference to those observed in the expression of *Hs*BR. The molecular reasons remain unclear but the differences elucidated here for the post-translational period seem to be key to understanding why folding and reconstitution with the chromophore retinal was successful for *Hs*BR but not for the other microbial rhodopsins.

## Discussion

The result of the UV/vis spectroscopic investigations show that only *Hs*BR is correctly folded upon *in-vitro* expression while the other tested microbial rhodopsins *Cr*ChR2, *Hs*SRI, and *Hs*SRII fail to establish tertiary structure. Our *in-situ* SEIRAS experiments provide evidence for successful insertion and folding of *Hs*BR by recording the dynamics of membrane insertion and folding *via* the intensities, contents, and peak positions of the α-helical component of the amide I band. As concluded from our previous experiments (Baumann et al., 2016; Harris et al., 2017), the initial 60 min refer to the co-translational period where the nascent peptide leaves the ribosome and simultaneously inserts into the membrane to form secondary structure. At this co-translational phase, the microbial rhodopsins *Cr*ChR2, *Hs*SRI, and *Hs*SRII all show an increase in α-helical content, suggesting the constant production and helical formation of the nascent peptide. Only in the case of *Hs*BR, the characteristic frequency upshift of the α-helical component takes place indicative of the insertion into the hydrophobic environment of the transmembrane domain and tertiary structure formation by aggregation of the transmembrane helices. On the other hand, the invariant peak positions observed in the other proteins suggest that the dielectric condition surrounding the nascent peptide does not alter during the co-translational period. *Cr*ChR2, *Hs*SRI, and *Hs*SRII fail to insert into the hydrophobic environment from hydrophilic interfacial region while they form their helical structures. We infer that the folding process of the nascent peptides of *Cr*ChR2, *Hs*SRI, and *Hs*SRII is stalled after 60 min at the level of partially folded structures that reside at the interface between the hydrophilic headgroups and the hydrophobic carbon chain of the lipids. Since penetration into the hydrophobic parts of the membrane may be essential for progression to form helical bundles, the nascent peptides remain at the interfaces and are not able to transform into proper tertiary structure. It is noted that only the apoprotein of *Hs*BR reconstitutes with the chromophore, while the other opsins fail to incorporate the retinal to establish the functional holoprotein. Since the retinal is highly hydrophobic, it can be safely anticipated that free retinal that is added to the *in-vitro* expression mix, enters deeply into the hydrophobic alkyl chains of the lipid bilayer. Hence, it would be a prerequisite for successful reconstitution that the nascent peptide is also able to enter the hydrophobic domain of the bilayer and orient its individual helices towards a perpendicular inclination with respect to the membrane surface for covalent binding with the retinal.

Hydropathy analysis of all four microbial rhodopsins predict that *Hs*BR reveals slightly higher driving force for insertion into the hydrophobic domain from the amphiphilic interfacial region of the lipid head group compared to *Cr*ChR2, *Hs*SRI, and *Hs*SRII. The hydropathy plot in the Octanol-Interfacial (Oct-IF) scale (Supplementary Information S3; Supplementary Figure S6) shows that the total Gibbs free energies for the peptide segment that preferably insert into the hydrophobic region are −18.13 (*Hs*BR), −13.94 (*Cr*ChR2), −12.22 (*Hs*SRI), and −10.91(*Hs*SRII) kcal/mol, respectively (Jayasinghe et al., 2001). It should be noted that the Gibbs free energy at the segment around helix G (residues 209–237) of *Hs*BR, which includes Lys216 that forms covalent linkage with the retinal in a fully matured protein, is rather smaller (−0.51 kcal/mol for *Hs*BR) than the other helical segments of helices A–F. Therefore, Lys216 in the G helix by itself may not be able to associate with the retinal molecule that most probably resides in the hydrophobic domain. However, it has been suggested that helices F and G are formed due to the interaction with helices A–E and pulled into the hydrophobic region (Booth, 2000). Slightly larger values in the total Gibbs energy of *Hs*BR is advantageous to pull the helical segments deeper into the hydrophobic domain and that facilitates the association between Lys216 and the retinal molecule. We infer that the association with retinal further increases hydrophobicity in the surrounding of the nascent peptide to support tertiary structure formation in the hydrophobic region. The importance of the association of the apoprotein with retinal is supported by our recent observation that omitting retinal during *in-vitro* expression also led to impaired folding of *Hs*BR (Baumann et al., 2016). It should be noted that the IR spectra of *Hs*BR in the absence of retinal exhibits many misfolded components attributed to bends or aggregates that differ from the spectral features of *CrChR2*, *Hs*SRI, and *Hs*SRII. We infer that the presence of pre-adsorbed retinal also changes the fluidity of the lipid bilayer, as the retinal molecule itself behaves similarly to cholesterol, which facilitates the insertion of a nascent polypeptide. It has been suggested that the physical properties of the lipid bilayer significantly affect the folding of many membrane proteins (Findlay and Booth, 2006).

In the case of *Cr*ChR2, *Hs*SRI, and *Hs*SRII, the weaker driving force to sink into the hydrophobic domain may hamper the association with the retinal halfway through the folding process. Although *Cr*ChR2, *Hs*SRI, and *Hs*SRII form a similar amount of α-helical structures as *Hs*BR during the co-translational insertion, further development into the functional tertiary structure was prevented.

In conclusion, the analysis of the *in-situ* recorded SEIRA spectra of four cell-free expressed microbial rhodopsins revealed that the non-functional production of *Cr*ChR2, *Hs*SRI, and *Hs*SRII does not take place on the level of transcription or translation but is due to a misfolding process that happens after translation. All proteins show successful secondary structure formation during the initial co-translational production period. However, the nascent peptides of *Cr*ChR2 and both sensory rhodopsins do not possess a sufficient driving force for insertion into the hydrophobic transmembrane domain. Our study demonstrates that applying SEIRA spectroscopy to track cell-free expression has potential not only for fundamental studies of membrane protein folding but also enables us to follow each peptide’s progress and to identify the point of failure. In this way, critical steps in the expression of membrane proteins can be identified and optimized to achieve higher expression rates or to provide remedies in case of malfunction.

## Data Availability

The original contributions presented in the study are included in the article/Supplementary Material, further inquiries can be directed to the corresponding authors.
