# Supplementary material for: Monitoring the Progression of Cell-Free Expression of Microbial Rhodopsins by Surface Enhanced IR Spectroscopy: Resolving a Branch Point for Successful/Unsuccessful Folding
Source: Front Mol Biosci. 2022 Jul 14;9:929285. doi: 10.3389/fmolb.2022.929285 (PMC9329800; doi:10.3389/fmolb.2022.929285)
Supplement: Supplementary file 1 [file Table1.docx]

Supplementary Material

Monitoring the progression of cell-free expression of microbial rhodopsins by Surface Enhanced IR spectroscopy: Resolving a branch point for successful/unsuccessful folding

**Kenichi Ataka^1^, Axel Baumann^2^, Jheng-Liang Chen^2^, Aoife Redlich^1^, Joachim Heberle^1^, and Ramona Schlesinger^2^**

^1^Experimental Molecular Biophysics, Department of Physics, Freie Universität Berlin, Berlin, Germany

^2^Genetic Biophysics, Department of Physics, Freie Universität Berlin, Berlin, Germany

# Supplementary Information SI-1: Cell-free expression of microbial rhodopsins at 37 °C

The cell-free expression of all proteins in the main text had been handled at room temperature (25 °C ± 2°C), in order to establish consistency with the IR measurement. However, this temperature is not optimal for the transcription/translation, which the manufacturer defines at 37 °C. This may lead to a question if the failures in cell-free expression of *Cr*ChR2 and sensory rhodopsins arise from these conditional differences. Thus, we had made the cell-free expression at 37 °C as a control. The results are shown in figure S1. Figure S1A shows Eppendorf caps with the cell-free expression attempts for each of the various microbial rhodopsins. The results are very similar to the cell-free expression at 25 °C, except that the expression mixture for *Hs*BR at 37 °C is more intense red than at 25 °C (figure 1A), which seems to be related to higher functional expression of the protein. The other mixtures remain yellow, comparable to the negative control and expressions at 25 °C (figure 1A). Figure S1B shows the UV/Vis spectra of the translation mixtures, cleared by centrifugation, in the range of 300 nm to 700 nm. The spectral features of all proteins are almost identical to those at 25 °C, except that the spectrum of the expression mixture for *Hs*BR shows almost 2 times higher absorption at 560 nm from the chromophore in the holoprotein in comparison to 25 °C. This result suggests that raising the expression temperature to 37 °C is most probably not a remedy for the failed expression of *Cr*ChR2 and sensory rhodopsins, although the rise in temperature significantly improves the expression yields of *Hs*BR.


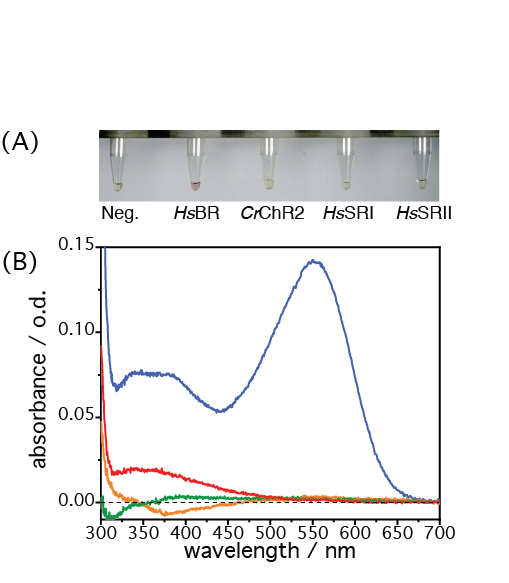


**Figure S1.** (A) The resultant solutions from the cell-free expression treated at 37°C after 8 hours. From left to right: Negative control (no DNA), bacteriorhodopsin (*Hs*BR), channelrhodopsin II *(Cr*ChR2), sensory rhodopsin I (*Hs*SRI), and sensory rhodopsin II (*Hs*SRII). (B) UV/visible absorption spectra of the resultant solutions of *Hs*BR (blue), *Hs*SRI(green), *Hs*SRII (red) and *Cr*ChR2 (orange). The solution with the negative control was used for the reference spectrum to compensate absorption from the free retinal in the solution and other components.

# SI-2: Data treatment

## Baseline correction

The observed raw spectra show a significant baseline drift compared to the signal intensity due to instability of the spectrometer for long measurement times (7 to 10 hours) (figure S2A). The baseline drift was corrected by the single point baseline correction at 1800 cm^-1^. The representative spectra after the baseline correction procedure are shown in figure S2B.


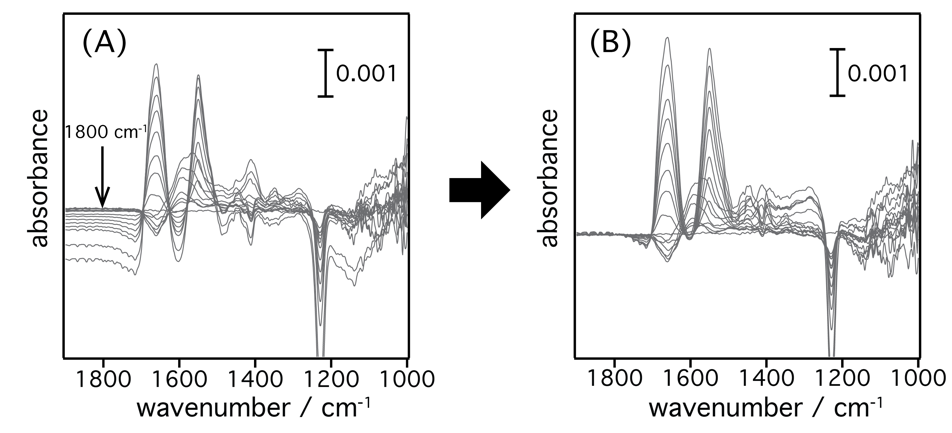


**Figure S2.** The baseline correction procedure. (A) A set of raw spectra of *Hs*BR before the baseline correction are shown. (B) Resulting spectra after single point baseline correction at 1800 cm^-1^

## Background subtraction

Some of the observed raw spectra, especially in the early stages of the nascent polypeptide insertion, show an overlay of the increasing positive bands from the insertion of the nascent polypeptide with the negative bands from the desorption of the non-specifically membrane-bound protein species of the cell-free expression mixture. Such an overlay is shown exemplary in Figure S3A in the time frame of 4.5 minutes to 18 minutes after starting the translation by DNA addition.


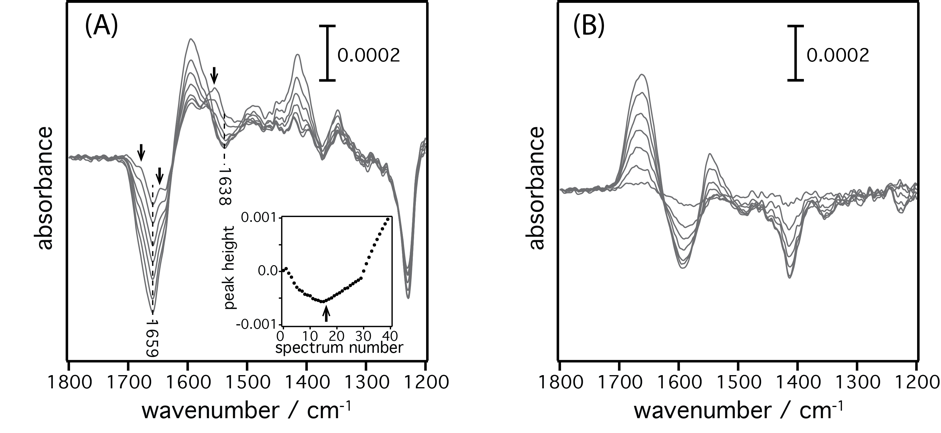


**Figure S3.** The background subtraction procedure. (A) A set of the baseline corrected spectra (*Hs*BR) before the background subtraction are shown. The spectra were chosen from those measured between 4.5 minutes (spectrum number 18) and 18 minutes (spectrum number 30). The inset box is a plot of the peak height at 1659 cm^-1^ versus the spectrum numbers. (B) the result spectra after the background subtraction of (A). The spectrum number 17 was chosen as the background.

The presence of negative bands is inconvenient for the peak fitting process because the overlay of negative and positive bands obscures the peak position of individual peaks and, as a consequence, leads to failure of the band fitting. Therefore, it is necessary to remove the negative peak contribution from the range of the positive peaks by subtracting with an appropriate reference spectrum. In order to find the relevant reference spectrum, heights of the negative peaks at 1659 cm^-1^ are plotted over the spectra numbers in the inset of figure S3A (the spectra numbers are defined in order of measurement with consecutive time intervals 10 s, 60 s, 5 min., 10 min., and 30 min. with 15 spectra accumulated for each interval). The peak height decreases in the first 16 spectra, then starts to increase. This increase is due to contribution from increases in the overlaid positive bands on top of the negative band. We chose spectrum #17, in which the peak height at 1659 cm^-1^ is at its minimum and indicates the least contribution from the overlaid positive bands, as the reference spectrum for subtraction. The result of the subtraction is shown in figure S3B. The overlap of the negative and positive bands at the amide I region (1620 - 1700 cm^-1^) are removed and the positive bands, which are attributed to the insertion of the nascent polypeptide, become clear. It should be noted that, as a result of the subtraction, the presence of the negative bands at ~1590 and ~1400 cm^-1^ are also additionally revealed. The origins of these bands are not clear. However, the spectral range of these bands is sufficiently deviated from the amide I and II bands so they do not hamper the fitting of amide bands, even when we include appropriate negative peaks at 1590 cm^-1^ during the fitting process.

## Peak fittings of the amide band

In order to carry out a suitable peak fitting on the amide I band, a number of secondary structure components should be properly deduced. We exploited the second derivative method to deduce the number of the components. Figure S4A shows the result of the second derivative of the spectrum observed at 63 minutes. In the second derivative spectrum (bottom), inflexion points in the amide band are observed as negative peaks at 1681, 1657, and 1640 cm^-1^ for amide I and 1552 and 1515 cm^-1^ for amide II, respectively. We assume that the former three peaks of amide I correspond to the positions of the secondary structural components and attempt the fitting based on these positions. The result of fitting is shown in figure S4B. The fitted peaks in amide I are shown in green (1681 cm^-1^), red (1657 cm^-1^), and blue (1640 cm^-1^). These peak positions correspond to bend/misfold, α-helix, and random structures, respectively. The cumulative of these peaks, shown as orange broken curves, corresponds to the observed spectrum (black curves) suggesting that the fitting works well.


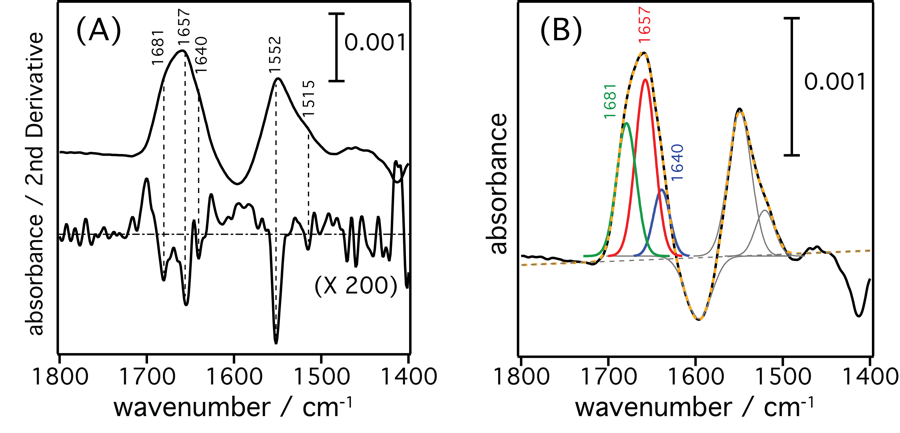


**Figure S4.** (A) The top spectrum of the *Hs*BR shows the nascent polypeptide at 63 minutes after DNA addition; the lower spectrum is the second derivative of the top spectrum. (B) The result of the peak fitting according to the peaks derived from the second derivative spectrum are given.

## Consecutive peak fitting for all spectra

Because the second derivative method only works for a spectrum that has a sufficiently good signal-to-noise ratio, detection of the secondary structure components by this method will fail for the spectra at the early insertion stage or those in the later stages of the measurement. The signal level of the spectra in the early stages is too low to distinguish between the inflexion points of the band and noise. In the case of the spectra obtained during the late stage of the measurement, subtle intensities from gaseous water vapor caused by atmospheric fluctuation during the long-time measurement overlay the amide I band. This leads to an artefact in the second derivative spectrum caused by the water vapor band which appears as a false inflection point. In order to avoid these problems, yet to achieve appropriate peak fitting for the early- and late-stage spectra, we employed the following ‘consecutive peak fitting’ approach.

Since we have measured the spectra sequentially in a relatively short period (10 sec - 30 min), the band features in one spectrum of the sequence is similar to those of the one before (figure S5). Therefore, the fitting parameters which are defined for an initial spectrum can then be used as a starting point for the fit calculated in the following spectrum. For example in the case of *Hs*BR, the spectra at 63 minutes (figure S3) gives the best fitting result because of its good signal-to-noise ratio. Then, this fitting result was used as the initial parameter for the fitting of the next spectrum at 68 minutes. This process was iterated through each sequential measurement. It should be considered that the spectral features and peak position of each component may change gradually over the long term, despite barely any discernible change in the spectrum of the nearest sequence. To manage such gradual changes in the spectra, we have imposed following constraint on the fitting:

1. The peak position of each component can change within the range of ±2 cm^-1^ for each iteration. This constraint gives freedom to change the peak of the spectral feature gradually.
2. The numbers of peaks are fixed to three components depending on the peak position. These secondary structure components are: a) bend/misfold, which appears at 1670-1685 cm^-1^, b) α-helix, which appears at 1648-1661 cm^-1^, and c) random, which appears at 1635 - 1645 cm^-1^.
3. The peak width of each component does not exceed more than 20 cm^-1^ to avoid overly broad peak shapes overlapping with the baseline.
4. All peaks are Gaussian.

**Figure S5.** The procedure for the consecutive peak fitting is shown. The black curve is a spectrum of the HsBR nascent peptide at 63 minutes (spectral number 40). The red and green curves are the spectra measured before (58 minutes) and after (68 minutes) the black spectrum, respectively.


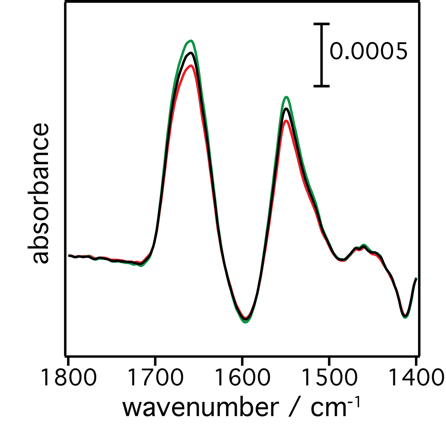


# SI-3: Hydropathy analysis of microbial rhodopsins

The hydropathy analysis of *Hs*Br, *Cr*ChR2, *Hs*SRI and *Hs*SRII had been made by the Membrane Protein Explorer (MPEx version 3.2) software provided on the homepage of the Stephen White Laboratory at UC Irvine (https://blanco.biomol.uci.edu/mpex/MPEXdoc.html). A Wimley-White scale was used for the calculation and set to partitioning from water to lipid bilayer. Amino acid sequences of *Hs*BR, *Hs*SRI and *Hs*SRII were taken from UniProt database (<https://www.uniprot.org>) with UniRef number of P02945, B0R633 and B0R6B0, respectively. The original amino acid sequence of truncated *Cr*ChR2 was taken from RCSB Protein Data Bank (6EID) and was modified to include an elongation at the C-terminus of ASHHHHHH, before the last 7 amino acids in the sequence, which is in agreement with the DNA plasmid used for expression.

The hydropathy plots of *Hs*BR, *Cr*ChR2, *Hs*SRI and *Hs*SRII are shown in figure S6. The plots are shown in the Wimley-White Oct-IF scale, which represents differences of the free energy of partitioning hydrocarbon region (Octanol scale) minus the free energy partitioning of the interface region (Interfacial scale) of the lipid bilayer. The segments with ΔG < 0 in the Oct-IF scale thermodynamically favor the insertion and formation of transmembrane helices in the hydrocarbon region relative to the partially folded state at the interface. The Oct-IF scale can therefore distinguish segments of the protein’s sequence which will form secondary structure. In Figure S6, these segments are identified by solid black lines running parallel to the x-axis for each protein. The known transmembrane helical segments derived from structural data are depicted as grey lines above the zero-line for comparison. The predicted and known segments correspond relatively well, except for the D helix (the 4^th^ grey line from left in figure S6A). This is due to the fact that Asp115 in the D helix of *Hs*BR or relevant Asp or Glu groups in other microbial rhodopsins are calculated in their charged (de-protonated) state. When these Asp or Glu groups are assumed to be in the protonated state, the D helix segment is predicted to be stable (Data not shown).


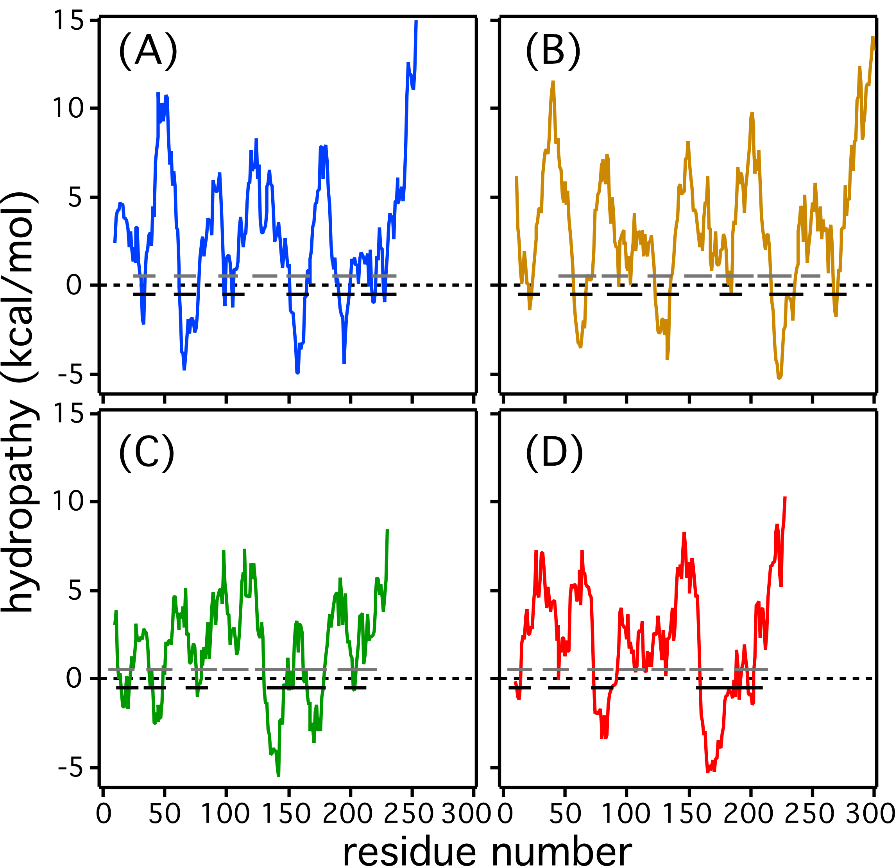


**Figure S6.** Hydropathy analysis of microbial rhodopsins (A) *Hs*BR, (B) *Cr*ChR2, (C) *Hs*SRI, and (D) *Hs*SRII. Presented Octanol-Interface scale analyses transfer free energetics from the interfacial region at the lipid head group to the transmembrane region of the hydrophobic tails. The black lines below the zero-line denote the residues predicted to form helical segments in the calculation, while the grey lines above the zero-line denote the known seven α-helical transmembrane segments.

The calculated Gibbs free energies of the predicted segments are summarized in table S1. Since the predicted segments prefer to be in the hydrocarbon region rather than the interface, the sum of these free energies of each protein is relevant to a driving force to place the nascent peptides, which initially reside at the interface after the co-translational period into the hydrophobic domain. Note that some of the calculated results could not resolve contributions from individual helices, as seen in the case of the E-F helices of *Hs*SRI and the F-G helices of *Hs*SRII, instead returning the sum of the free energy from these helices. A comparison among the total Gibbs free energies reveals that *Hs*BR provides slightly higher values than the others. This result suggests that *Hs*BR is more prone to sink into the hydrophobic domain to proceed tertiary structure formation than the other rhodopsins.

| Relevant TM helices | ***Hs*BR** | | ***Cr*ChR2** | | ***Hs*SRI** | | ***Hs*SRII** | |
| --- | --- | --- | --- | --- | --- | --- | --- | --- |
|  | ΔG  (kcal/mol) | Residue number | ΔG  (kcal/mol) | Residue number | ΔG  (kcal/mol) | Residue number | ΔG  (kcal/mol) | Residue number |
| A | -2.21 | (24-42) | -1.4 | (12-30) | -1.68 | (11-29) | -1.18 | (4-22) |
| B | -4.8 | (57-75) | -3.52 | (84-112) | -2.48 | (33-51) | -0.05 | (36-54) |
| C | -1.25 | (96-114) | -1.06 | (124-142) | -1.02 | (67-85) | -3.37 | (71-89) |
| D | ND |  | -4.23 | (175-193) | ND |  | ND |  |
| E | -4.94 | (148-166) | -0.62 | (175-193) | -6.38 | (133-180) | ND |  |
| F | -4.42 | (186-204) | -3.46 | (215-243) |  |  | -6.31 | (156-210) |
| G | -0.51 | (209-237) | -1.77 | (260-278) | -0.66 | (195-213) |  |  |
| Total | -18.13 |  | -13.94 |  | -12.22 |  | -10.91 |  |

**Table S1.** Summary of the results from the hydropathy calculation of microbial rhodopsins in the Oct-IF scale. Residue numbers denote the predicted segments relevant to the known seven transmembrane helices A-G
